# Research on Surface Treatment and Interfacial Bonding Technology of Copper–Polymer Direct Molding Process

**DOI:** 10.3390/ma14112712

**Published:** 2021-05-21

**Authors:** Qingsong Cao, Rongsheng Guo, Feng Yang, Jian Rong, Guanghong Hu

**Affiliations:** 1 Department of Plasticity Technology, Shanghai Jiao Tong University, Shanghai 200030, China; cqs515818wsz@sjtu.edu.cn (Q.C.); guo_rs@foxmail.com (R.G.); yang_feng@sjtu.edu.cn (F.Y.); 2 Qingdao Huatao Automobile Mould Co. Ltd., Qingdao 266032, China; qdpmrj@163.com

**Keywords:** surface treatment, copper, anodic oxidation, microstructure, injection molding, bonding strength

## Abstract

To realize the connection of copper and Polyphenylene sulfide (PPS) by metal–polymer direct molding, this paper combined anodic oxidation and chemical corrosion to treat the surface of copper, and carried out the injection molding experiment. An orthogonal experimental arrangement was used to identify the optimal electrolyte and etching solution for preparing a microstructure on a copper surface. The bonding and fracture mechanisms of the copper–polymer assembly were investigated through injection molding experiment and SEM technology. The results revealed that the phosphoric acid concentration had the most significant effect on the microstructure quality and etching solution containing 20% phosphoric acid produced a uniform microstructure with 25.77% porosity and 5.52 MPa bonding strength. Meanwhile, SEM images of the interface from bonding to fracture in the copper–polymer assembly indicated a well-filled polymer in the microstructure with a mainly cohesive fracture mode.

## 1. Introduction

Due to the demand for lightweight products, the performance of a single material can no longer meet the needs of the market, and the composite molding of multiple materials has become a trend. Composite molding mainly includes composite molding between dissimilar metals, such as explosive welded joints between Al and Cu [1,2], and metal–polymer composite molding, such as stainless steel and PPS [3]. This paper focus on the latter and there are three types of metal–polymer composite molding technologies: multi-component injection molding, metal–polymer bond synthesis, and metal–polymer direct molding [4,5]. The reason metal–polymer direct molding is widely used is that it offers the advantages of short production cycle, low environmental sensitivity, no requirement on the bonding surface, and high bonding strength [6].

Literature has shown that the formation of microstructure on metal surface is the key to the quality of metal–polymer assemblies. The bonding strength rises with the increase of roughness of metal surface [5,7]. Anodic oxidation is a simple and common method to prepare microstructure on metal surface, which is widely applied to aluminum [8,9,10,11,12], stainless steel [3,13], titanium [14,15], tin [16], and other metals. The basic principle is to use acid electrolyte to corrode micropores on metal surface. However, anodic oxidation cannot be employed to copper because copper is difficult to corrode by acid electrolyte, which limits the application of copper in metal–polymer direct molding. Copper is a common and excellent metal that offers advantages such as good electrical conductivity, thermal conductivity, oxidation resistance, corrosion resistance, and easy plastic processing. Therefore, copper–polymer assembly obtains widely attention in industrial production. As for the investigation of copper surface treatment, Kim et al. [17] adopted thermal precipitation technology to treat the copper, and found that hydrogen plasma treatment significantly enhances the bonding strength between the copper and polymer by improving the hydrophobicity of metal. However, this method is complex and expensive, and difficult to be widely used in industry.

To overcome these problems, anodic oxidation and chemical corrosion are combined to prepare microstructure on the surface of copper in this work. First, the oxide film is formed on the surface of copper by electrolysis, and then the copper is immersed in the etching solution to make chemical reaction between the oxide film and the etching solution, to prepare the microstructure on the surface of copper. At the same time, to obtain the optimal surface treatment solutions and the best concentration ratio, this study experimentally investigated different types of electrolytes and etching solutions. Furthermore, the injection molding experiments were carried out to prepare the copper–polymer assemblies. The bonding strength of each assembly was tested, and the microstructure was analyzed by SEM.

## 2. Surface Treatment of Copper

### 2.1. Materials for Surface Treatment

This study used TU1 oxygen-free copper with purity of 99.97%. The oxygen content is not more than 0.003%, and the total impurity content is not more than 0.03%. The material was laser cut into a cuboid with dimensions of 40 mm × 5 mm × 4 mm. The copper surface was cleaned using chemical compounds such as anhydrous ethanol, acetone, and sodium hydroxide. Sodium hydroxide, sodium carbonate, and sodium molybdate were used in the electrolysis of copper. Solution 1: Concentrated hydrochloric acid and sodium chloride solutions, solution 2: Concentrated phosphoric acid and sodium dihydrogen phosphate solutions, and solution 3: Concentrated nitric acid and sodium nitrate solutions, were used for the corrosion of copper [18]. All the aforementioned chemicals were provided by Sinopharm Group in Shanghai, China.

### 2.2. Experimental Plan for Surface Treatment

**Figure 1:**
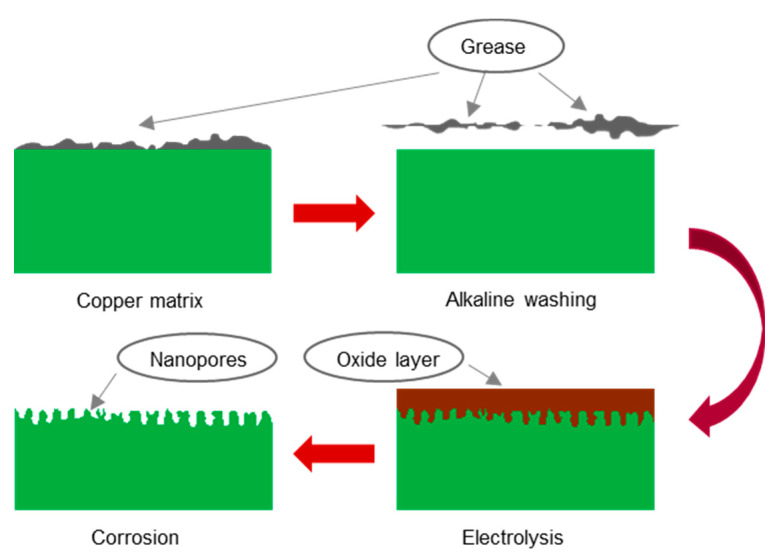
Schematic representation of surface treatment procedure for copper.

The copper surface treatment process was similar to that of aluminum and stainless steel, except that an electrolysis process was applied prior to chemical corrosion. Figure 1 displays the entire experimental process.

(a) Pretreatment: Before anodic oxidation, the copper surface needed to be cleaned to remove oil stains, rust stains, and dust. First, the copper was rinsed with tap water to remove dust. Second, the copper was rinsed with alcohol and acetone for 5 min to remove oil stains. Third, the copper was rinsed with a 1% sodium hydroxide solution for 3 min for further oil stain removal. Finally, the copper sheet was washed with deionized water and dried.

(b) Preparation of the electrolyte and etching solution: After consulting the relevant literature [19,20], Two different electrolyte solutions were prepared: one was a mixture of sodium hydroxide and sodium molybdate, and the other was a mixture of sodium carbonate and sodium molybdate. Three etching solutions were prepared: one was a mixture of hydrochloric acid and sodium chloride, another was a mixture of phosphoric acid and sodium dihydrogen phosphate, and another was a mixture of nitric acid and sodium nitrate.

(c) Anodic oxidation: The copper matrix was used as the anode, and a graphite carbon rod was used as the cathode. Both the anode and the cathode were partially immersed in the electrolyte, and the distance between them was approximately 10 cm. Electrolysis was performed at 15 V for 5 min.

(d) Chemical corrosion: First, the anodized copper sample was immersed in the etching solution. The solution was then stirred gently. Finally, the corroded copper sample was removed, washed with deionized water, and dried.

### 2.3. Characterization of the Surface Treatment

A JEOL JSM-7800F Prime super resolution field-emission scanning electron microscope (SEM) (JEOL, Tokyo, Japan) and a Thermo Scientific NORAN™ System 7 energy-dispersive X-ray spectrometer (EDS) (Thermo Scientific, Waltham, MA, USA) were used to evaluate the surface morphology of copper after surface treatment at a high acceleration voltage of 5 kV. EDS was performed at an acceleration voltage of 15 kV to determine the composition and content of the copper surface microstructure.

**Figure 2:**
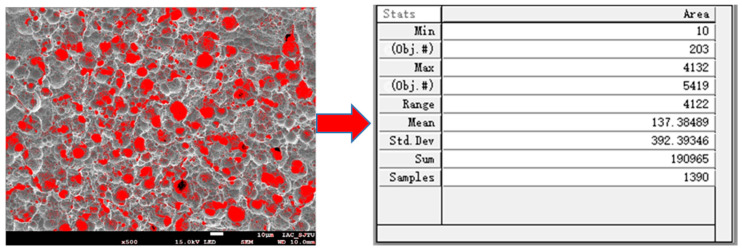
Statistics of porosity using Image-Pro Plus software.

As shown in Figure 2, Image-Pro Plus 6.0 software (Media Cybernetics, Rockville, Maryland, USA) was used to calculate the processed copper surface porosity, and the porosity was used to judge the quality of the copper surface morphology.

### 2.4. Phenomena of Surface Treatment in Copper

**Figure 3:**
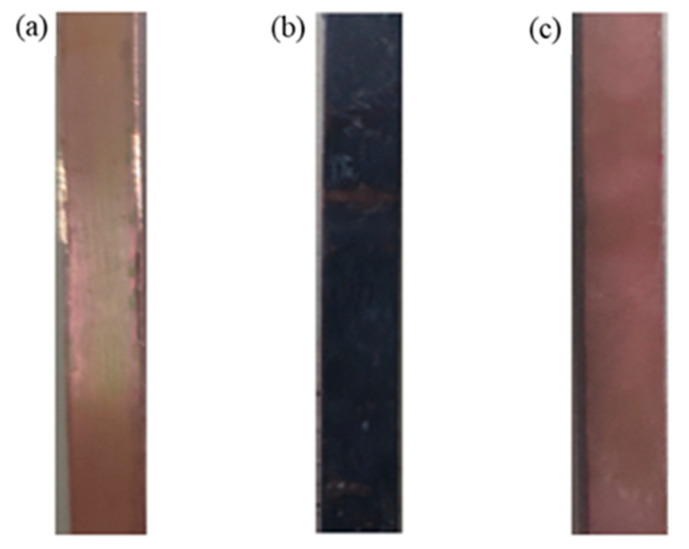
The color of copper surface at different stages. (**a**) before treatment; (**b**) after oxidation; (**c**) after chemical corrosion.

During the anodic oxidation, the energized copper sheet began to react violently, the area around the anode copper sheet gradually turned light blue, and a large amount of gas was generated near the cathode carbon rod. This occurred because the anode copper sheet lost electrons and was oxidized into copper ions, whereas the cathode water molecules gained electrons and decomposed into *H_2_* and *OH^−^*. Gradually, the anode copper sheet changed color from purplish red with a metallic luster to gray because as the reaction progressed, the *Cu^2+^* concentration in the electrolyte continued to increase and *Cu^2+^* combined with *OH^−^* to precipitate *Cu(OH)_2_.* As the electrolysis reaction was exothermic, when the electrolyte temperature reached 60–80 °C, the *Cu(OH)_2_* precipitate decomposed into black *CuO* and water. The current density remained basically stable throughout the process; specifically, it was proportional to the electrolysis voltage and was independent of other parameters such as electrolyte concentration and type. Figure 3a,b show the color change of the copper surface.

During chemical corrosion, the anodized copper sheet was immersed in the etching solution. The reaction between the copper sheet and the etching solution was relatively gentle. The color of the copper surface gradually changed from grayish black to the original purplish red, and the etching solution near the copper surface turned light blue. If the solution is uniformly stirred, then no significant changes will occur because a small amount of *CuO* attached to the copper surface should react with the etching solution to generate a small amount of *Cu^2+^*. Figure 3c shows the color of the copper surface after chemical etching. The following chemical reactions occurred during the aforementioned processes.

(1) $$Cu-2e^{-}=Cu^{2+}\;Cu^{2+}+2OH^{-}=Cu{\left(OH\right)}_{2}↓\;Cu{\left(OH\right)}_{2}≜CuO+H_{2}O\;2H_{2}O+2e^{-}=H_{2}↑+2OH^{-}\;CuO+2H^{+}=Cu^{2+}+H_{2}O$$

**Figure 4:**
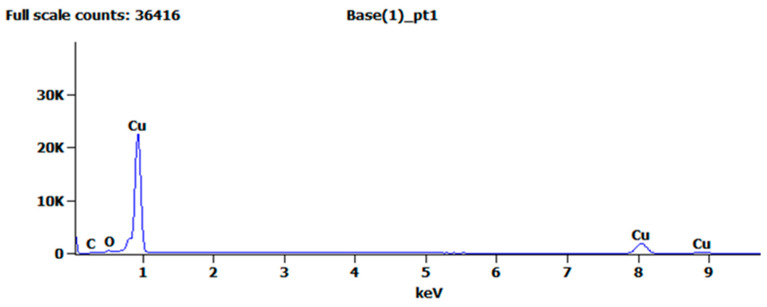
EDS analysis of the specimen after chemical corrosion.

Figure 4 shows the EDS analysis of the treated copper surface, and Table 1 shows the elemental composition of the copper surface. These results indicate that compared with the pure copper before surface treatment, the metal content basically remained unchanged, whereas the oxygen and carbon contents increased slightly. The increase in oxygen content was due to a small amount of copper oxide residue on the copper surface, and the increase in carbon content was due to copper oxide reacting with carbon dioxide in the air to form a basic copper carbonate. The EDS analysis results can also explain the experimental phenomenon of the copper surface turning grayish black during electrolysis. The aforementioned analysis demonstrated that after anodic oxidation and chemical corrosion, no residue remained on the copper surface.

### 2.5. Selection of the Electrolyte and Etching Solution for Surface Treatment

To determine the formulation of electrolytes and etching solutions, this study de-signed six experimental schemes to investigate two types of electrolytes and three types of etching solutions. Table 2 shows the electrolyte and etching solution ratios in each experimental scheme.

**Figure 5:**
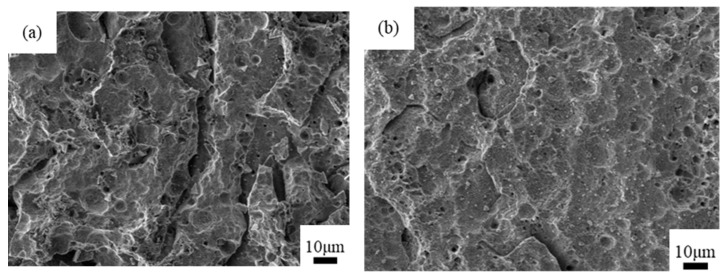

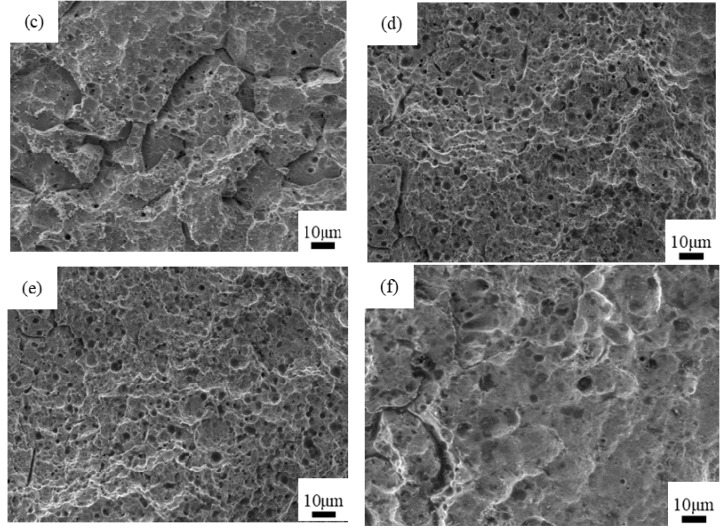
Micromorphology of the copper surface by SEM in six schemes. (**a**) Scheme 1; (**b**) Scheme 2; (**c**) Scheme 3; (**d**) Scheme 4; (**e**) Scheme 5; (**f**) Scheme 6.

Surface treatment experiments were performed according to the six experimental schemes listed in Table 2, and the corresponding microstructure morphology of the copper surface was ascertained. These six schemes were intuitively compared using SEM images, as shown in Figure 5.

Dividing Figure 5 into two groups—(a,b,c) and (d,e,f)—reveals that when the same electrolyte was used, the corrosion effect of the etching solution containing phosphoric acid was better than that of the etching solution containing hydrochloric acid and nitric acid. Dividing Figure 5 into three groups—(a,d), (b,e), and (c,f)—reveals that when the same etching solution was used, the oxidation effect of the electrolyte containing sodium carbonate was better than that of the electrolyte containing sodium hydroxide. At the same time, the corrosion effect in Figure 5e is better than others, where the electrolyte containing sodium carbonate and the etching solution containing phosphoric acid were used. Therefore, in this study, the electrolyte containing sodium carbonate and the etching solution containing phosphoric acid were selected for evaluating the microstructure formation process on the copper surface.

### 2.6. Relationship between Surface Treatment Process and Morphology of Copper Surface

Based on comparisons of the above six experimental schemes, the electrolyte containing sodium carbonate and the etching solution containing phosphoric acid were finally selected for use. However, the specific concentration ratio remains uncertain; this issue requires further experimental study. In this regard, porosity was selected as the objective function, and the phosphoric acid concentration, sodium dihydrogen phosphate concentration, corrosion time, electrolysis time, sodium carbonate concentration, and electrolysis voltage were selected as six influencing factors. The L_25_(5^6^) orthogonal table was designed to arrange the experiment. There were 25 groups of experiments. In each group of experiments, one sample was selected, and four photos (with 1000×, 2000×, 5000× and 10,000× magnifications) were selected for observation, and the porosity of the copper surface microstructure obtained through the 25 groups of experiments was determined. Table 3 lists details of the orthogonal experimental scheme, experimental results, and range analysis.

**Figure 6:**
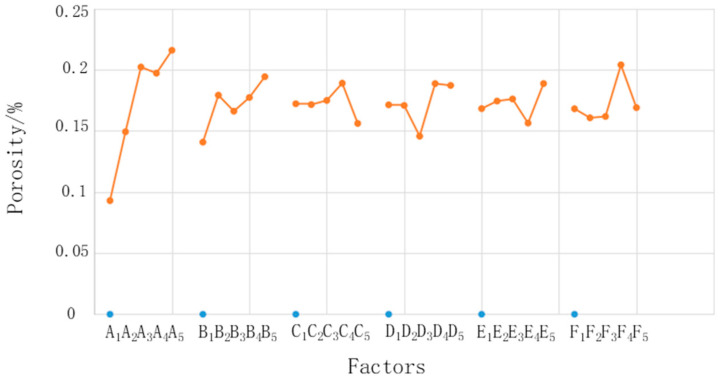
Relationships between the factors and porosity.

Where *K*_1_–*K*_5_ are the average values of porosity of the microstructure on the copper surface under different levels. The *K* value was calculated as follows.

(2) $$K_{ij}=\frac{1}{n}\sum_{m=1}^{n}p_{ji,m}$$

where *i* is the level, *j* is the factor, *n* is the level number of each factor (in this experiment, *n* = 5), *m* is the number of experimental groups under the same factor and same level, and *p* is the porosity. Taking *K*_12_ as an example, the *K* value is the mean of five porosity values corresponding to the second factor (concentration of sodium dihydrogen phosphate) and the first level (1%). The range *R* is the difference between the maximum value and the minimum value of *K*_1_–*K*_5_ for each factor. Figure 6 shows relationships between the factors and porosity. It shows that the factors most influenced the microstructure, in descending order, were phosphoric acid concentration, sodium dihydrogen phosphate concentration, electrolysis voltage, electrolysis time, chemical corrosion time, and sodium carbonate concentration. The phosphoric acid concentration had the largest range and had a much larger influence than the other factors. Although the other factors had different ranges, the difference was small; therefore, this study focused on the influence of only the phosphoric acid concentration.

The most important step in the microstructure preparation process was to corrode the oxide film formed by anodic oxidation. The acidity of the etching solution plays a very important role during the corrosion process. If the acidity of the etching solution is too strong, in addition to reacting with the oxide film, it will also react with copper and destroy the microstructure. However, if the acidity of the etching solution is not strong enough, then it will not fully react with the oxide film and only some pits or large-scale microstructures will form on the copper surface. The phosphoric acid concentration directly determines the strength of its acidity and therefore has a particularly significant influence on the microstructure. By contrast, other factors have less influence on the chemical corrosion process and on the formation of the final microstructure.

**Figure 7:**
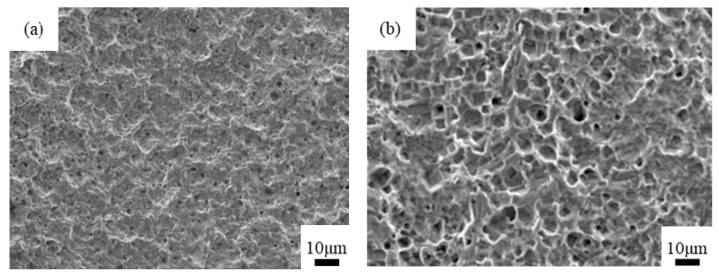

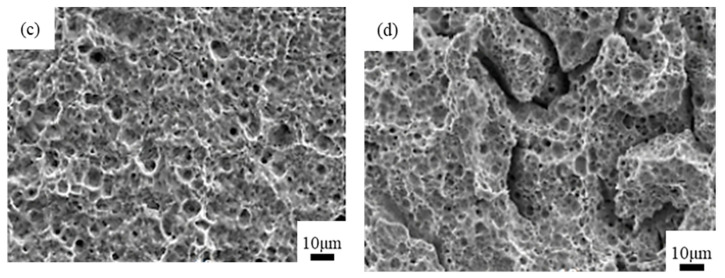
SEM images of copper surface treated with different concentrations of phosphoric acid. (**a**) 16%; (**b**) 18%; (**c**) 20%; (**d**) 22%.

A single factor and single target experiment was performed to study the influence of the phosphoric acid concentration in the etching solution on porosity. The phosphoric acid concentration was set to 16%, 18%, 20% and 22%, and other parameters were set according to the optimal combination (B_5_, C_4_, D_4_, E_5_, F_4_); specifically, the sodium dihydrogen phosphate concentration was 5%, corrosion time was 28 min, electrolysis time was 18 min, sodium carbonate concentration was 25%, and electrolysis voltage was 18 V. After the surface treatment of copper, the micromorphology of the copper surface was evaluated. The result is displayed in Figure 7.

Figure 7 shows that when the phosphoric acid concentration was 16%, only some uneven structures formed from corrosion on the copper surface; although the surface was much rougher after corrosion, micropores did not form. When the phosphoric acid concentration increased to 18%, many porous structures with an average diameter of 1800–2000 nm formed on the copper surface. At a phosphoric acid concentration of 20%, the micropores that formed on the copper surface were denser and more uniform and the average diameter was smaller, at 800–1500 nm. Finally, when the phosphoric acid concentration increased to 22%, the size of the micropores on the copper surface was less affected, and some gully structures formed. A phosphoric acid concentration of 20% was seen to produce microstructures with the best size and uniformity on the copper surface. Five samples with a phosphoric acid concentration of 20% were selected for analyzing the pore size statistics, and the average pore size was calculated to be 1200 nm, as shown in Table 4.

## 3. Bonding Strength of the Copper–Polymer Assembly

### 3.1. Materials for the Injection Molding Experiment

This study used PPS as the polymer. PPS is a black granular material with an average molecular weight of 4000–5000 and a density of 1.3–1.8 g/cm^3^. Table 5 lists the main physical parameters of PPS.

### 3.2. Equipment and Molds for the Injection Molding Experiment

This study used a Sumitomo SE180DU injection molding machine (SUMITOMO, Tokyo, Japan); Table 6 lists its basic parameters. This machine was equipped with heating oil pipes on both the fixed and movable mold sides and used oil temperature heating to realize rapid heating and cooling of the mold. Before each injection molding, the mold needed to be heated to a suitable predetermined value.

### 3.3. Process Flow of the Injection Molding Experiment

First, the PPS and the surface-treated copper sheet were dried and preheated. Next, the metal sheet was placed in the mold cavity and left to stand for approximately 10 s. The injection molding process commenced when the temperatures of the metal substrate and the mold were almost the same to ensure that PPS could fully fill the micropores on the metal substrate surface. The injection molding process parameters, shown in Table 7, were set according to the PPS parameters.

**Figure 8:**
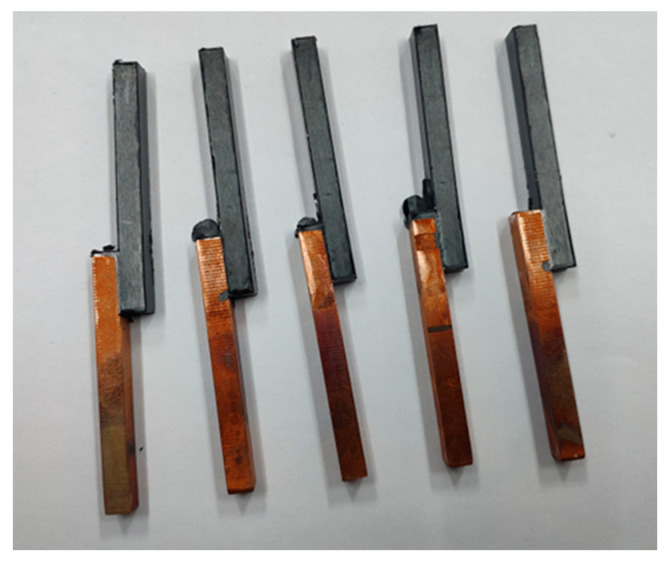
Schematic representation of the copper-PPS assemblies.

The injection molding experiment was performed to obtain the copper-PPS assembly. The bonding surface of copper and PPS was a 5 mm × 10 mm plane. Figure 8 shows schematic representation of the assemblies.

### 3.4. Influence of the Molding Process on the Bonding Strength of the Copper-PPS Assembly

The most important parameter in the metal–polymer direct molding technology is the bonding strength of the metal–polymer assembly. In this study, referring to international standard ISO-19095(plastics-evaluation of the adhesion interface performance in plastic-metal assemblies), a tensile experiment was performed using the Zwick Z020 universal material testing machine to measure the bonding strength of the copper-PPS assembly. The two ends of the assembly were clamped in the thickness direction before tensile measurements were performed. The maximum ultimate strength of the bonding surface of the assembly was obtained through the tensile load-displacement curve.

**Figure 9:**
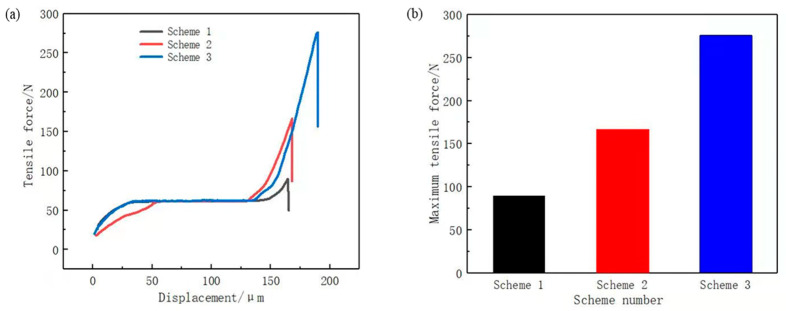
(**a**) tensile force curve; (**b**) maximum tensile force.

To verify the corrosion effect of the phosphoric acid and sodium dihydrogen phosphate etching solution, three sets of comparative experiments were designed to test the bonding strength of the assemblies formed under each scheme, as shown in Figure 9a.

Scheme 1: Chemical etching solution of hydrochloric acid with a concentration of 20%.

Scheme 2: Chemical etching solution of phosphoric acid with a concentration of 20%.

Scheme 3: Chemical etching solution of phosphoric acid with a concentration of 20% and sodium dihydrogen phosphate with a concentration of 5%.

Figure 9b displays the maximum tensile force. In ascending order, the scheme ranking according to stretching force was Scheme 1, Scheme 2, and Scheme 3. This is consistent with the abovementioned results of the etching solution selection, thus verifying that 20% phosphoric acid with 5% sodium dihydrogen phosphate is a suitable chemical etching solution. Considering that the bonding surface of copper and PPS was a 5 mm × 10 mm plane and the maximum tensile force of the Scheme 3 was 276.15 N, the bonding strength of Scheme 3 was calculated to be 5.52 MPa using Equation (3). The bonding strength was greater than 5 MPa, which basically meets the strength requirements of new energy vehicle battery connector.

(3) $$\tau =\frac{F}{A}$$

where *F* is the tensile force, *A* is the area of the bonding interface of the assembly, and *τ* is the shear stress (i.e., bonding strength) of the assembly.

### 3.5. Bonding Mechanism of the Copper-PPS Assembly

**Figure 10:**
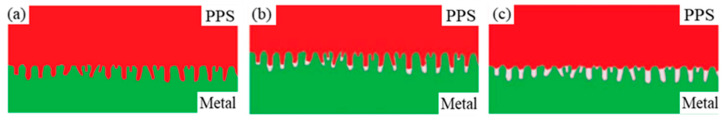
Schematic representation of three filling models. (**a**) completely filled; (**b**) partially filled; (**c**) unfilled.

The micromechanical connection between the metal and polymer refers to the mechanical anchor structure formed between the thermoplastic polymer flowing into the surface of the metal matrix and the microstructure. The bonding strength of the assembly also directly depends on the structure of the mechanical anchor. The bonding interface can be divided into three states according to the amount of molten polymer injected into the microstructure, as shown in Figure 10.

(a) Completely filled: The polymer completely fills the micropores on the metal surface and is thoroughly combined with the metal. This is an ideal model that is difficult to produce because the process conditions do not allow for the ideal state to be easily reached.

(b) Partially filled: The polymer partially fills the micropores on the metal surface. During injection molding, the air in the micropores cannot usually be removed, and the molten polymer cannot completely fill the micropores, leading to insufficient strength of the bond between the metal and the polymer. In practice, this type of bonding model is commonly produced.

(c) Unfilled: The polymer does not fill the micropores on the metal surface, and there is no mechanical anchor structure between the metal and the polymer. This is probably due to improper injection molding process parameters, such as insufficient preheating of the copper sheet and low mold temperature.

**Figure 11:**
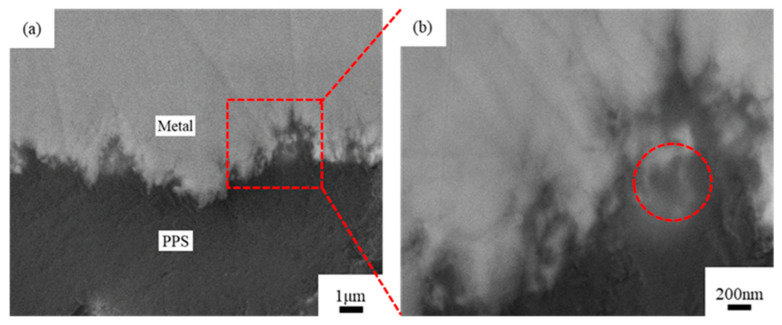
SEM images of metal-PPS bonding interface sections. (**a**) 5000× magnification; (**b**) 20,000× magnification.

Figure 11 presents SEM images of the bonding interface section of the copper-PPS assembly obtained through injection molding when 20% phosphoric acid and 5% sodium dihydrogen phosphate was used as the chemical etching solution.

Figure 11 shows that multiple layers of uneven network pores were present on the copper surface, and large pores contained small pores in a manner similar to a “coral reef” structure. PPS fully filled this “coral reef” structure to form an “anchor bolt” structure. However, a few pores had insufficient PPS filling, with the front end of the melted PPS cooling and stagnating before reaching the bottom of the pores, as indicated by the circle in Figure 11b. In this case, the filling effect can be further improved through optimization of the injection molding process and mold design.

### 3.6. Fracture Mechanism of the Copper-PPS Assembly

**Figure 12:**
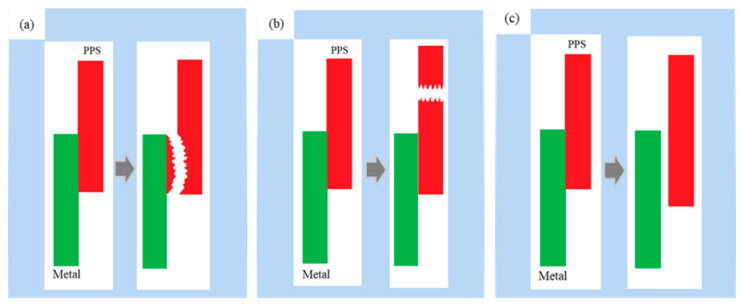
Schematic representation of three fracture models. (**a**) interface cohesive fracture; (**b**) polymer body fracture; (**c**) interface peeling fracture.

Fracture models of the metal–polymer assembly can be roughly divided into three types: interfacial cohesive fracture, polymer bulk fracture, and interfacial peeling fracture, as shown in Figure 12.

(a) Interface cohesive fracture: In this fracture model, the bonding surface is peeled off, and there are some polymer residues on the fracture surface on the metal side. When the assembly is stretched, if the increasing tensile force is greater than the strength of the “interlocking structure” formed at the interface but lower than the strength of the polymer body, interface cohesive fracture occurs.

(b) Polymer body fracture: The bonding surface of the metal and the polymer is intact; however, the polymer body is broken. When the assembly is stretched, if the increasing tensile force is greater than the strength of the polymer body but lower than the strength of the “interlocking structure” formed at the interface, then the polymer body fractures.

(c) Interface peeling fracture: The polymer is completely peeled from the metal surface, and there is no polymer residue on the metal side. This fracture model generally occurs when the polymer does not sufficiently fill the micropores on the metal surface or when no effective “interlocking structure” has formed between the micropores on the metal surface and the polymer.

**Figure 13:**
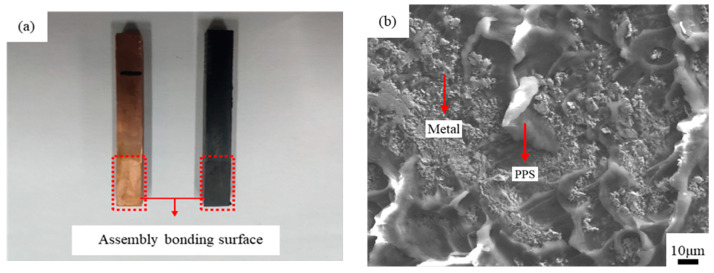
The fracture interface between copper and PPS. (**a**) schematic representation; (**b**) SEM image.

Figure 13a shows a schematic representation of the fracture interface between copper and PPS. After separation, a layer of residual PPS was observed on the copper surface. This PPS layer filled the micropores on the surface of the copper matrix in a molten state during injection molding and formed a micromechanical interlocking structure with the copper matrix after cooling. During the tensile test, under the action of the drawing force, the micromechanical interlocking structure between copper and PPS was destroyed, and part of the PPS remained on the copper surface. Figure 13b presents an SEM image of the fracture interface of the copper-PPS assembly.

Figure 13b shows that when the copper-PPS assembly fractured and separated, cohesive fracture occurred in the area where PPS remained on the copper surface because the molten PPS was fully injected into the micropores on the copper surface during injection molding and strong mechanical interlocking structures formed. During the tensile test, when the load exceeded the strength of the PPS material, the PPS fractured at the position where it was combined with copper, and part of it remained on the copper surface. In areas with no PPS residue or only a small amount of residue on the copper surface, interface peeling fracture or mixed fracture occurred between the copper and PPS because this area was not completely injected into the micropores during injection molding. No tight mechanical interlocking structure formed between copper and PPS. Therefore, when the assembly was broken, the PPS directly separated from the metal surface.

## 4. Conclusions

This study experimentally investigated copper surface treatments, the preparation process of a microstructure, and the bonding strength of a copper-PPS assembly. The following conclusions were drawn from the study:

(1) The microstructure of a copper surface was prepared using a surface treatment process that combined chemical and electrochemical methods. Sodium carbonate was found to be better than sodium hydroxide for the electrolyte, and phosphoric acid was found to be better than hydrochloric acid and nitric acid for the etching solution.

(2) The influence of various process parameters on the quality of the copper surface microstructure was studied through orthogonal experiments, and the phosphoric acid concentration was found to have the greatest influence, followed by the sodium dihydrogen phosphate concentration, electrolysis voltage, electrolysis time, chemical corrosion time, and sodium carbonate concentration. When the phosphoric acid concentration was set to 20%, a uniform microstructure with porosity of up to 25.77% could form on the copper surface.

(3) The copper and polymer were mainly connected through the formation of a micromechanical interlocking structure. The better the quality of the microstructure on the copper surface, the easier it was to fully inject the molten polymer into the microstructure during injection molding. In turn, the micromechanical interlocking structures between the copper and the polymer were closer and more robust, and the bonding strength of the assembly was higher. The injection molding experiment verified that the injection molding assembly could achieve the highest bonding strength of 5.52 MPa when 20% phosphoric acid with 5% sodium dihydrogen phosphate was used as the etching solution.

(4) SEM images of the bonding interface section and fracture surface of the copper-PPS assembly show that the bonding model of the sample obtained from the surface treatment process and injection molding process in this study was relatively similar to the ideal “completely filled” model. The fracture model was also relatively similar to the “interface cohesive fracture” model. These results indicate that the PPS filling in the copper surface microstructure is sufficient and that the copper surface treatment process and injection molding process are feasible for application.

## Figures and Tables

**Table 1:** Elemental composition and content of the microstructure.

Element.	*Cu*	*O*	*C*
Mass percentage/%	97.88	1.24	0.88

**Table 2:** Electrolyte and etching solution ratios in each experimental scheme.

No.	Electrolyte/wt. %			Etching Solution/wt. %					
	Sodium Hydroxide	Sodium Carbonate	Sodium Molybdate	Hydrochloric Acid	Sodium Chloride	Phosphoric Acid	Sodium Dihydrogen Phosphate	Nitric Acid	Sodium Nitrate
1	20	--	5	30	5	--	--	--	--
2	20	--	5	--	--	30	5	--	--
3	20	--	5	--	--	--	--	30	5
4	--	20	1	30	5	--	--	--	--
5	--	20	1	--	--	30	5	--	--
6	--	20	1	--	--	--	--	30	5

**Table 3:** Range analysis of orthogonal experiments.

No.	Phosphate/%	Sodium Dihydrogen Phosphate/%	Corrosion Time/Min	Electrolytic Time/Min	Sodium Carbonate/%	Voltage/V	Porosity/%
1	16	1	22	12	5	12	4.99
2	16	2	24	14	10	14	8.57
3	16	3	26	16	15	16	5.78
4	16	4	28	18	20	18	14.66
5	16	5	30	20	25	20	12.45
6	17	1	24	16	20	20	7.50
7	17	2	26	18	25	12	19.11
8	17	3	28	20	5	14	16.69
9	17	4	30	12	10	16	13.47
10	17	5	22	14	15	18	20.90
11	18	1	26	20	10	18	21.97
12	18	2	28	12	15	20	22.30
13	18	3	30	14	20	12	15.99
14	18	4	22	16	25	14	18.50
15	18	5	24	18	5	16	22.30
16	19	1	28	14	25	16	18.45
17	19	2	30	16	5	18	18.65
18	19	3	22	18	10	20	20.78
19	19	4	24	20	15	12	21.56
20	19	5	26	12	20	14	19.09
21	20	1	30	18	15	14	17.45
22	20	2	22	20	20	16	20.90
23	20	3	24	12	25	18	25.77
24	20	4	26	14	5	20	21.43
25	20	5	28	16	10	12	22.36
*K*_1_	0.0929	0.1407	0.1721	0.1712	0.1681	0.1680	
*K*_2_	0.1492	0.1791	0.1714	0.1707	0.1743	0.1606	
*K*_3_	0.2021	0.1660	0.1748	0.1456	0.1760	0.1617	
*K*_4_	0.1971	0.1772	0.1889	0.1886	0.1563	0.2039	
*K*_5_	0.2158	0.1942	0.1560	0.1871	0.1886	0.1689	
*R*	0.1229	0.0535	0.032943	0.0430	0.0323	0.0433	

**Table 4:** Porosity in five groups of experiments.

No.	1	2	3	4	5	Mean Value
Aperture/nm	800	1300	1100	1300	1500	1200

**Table 5:** Physical parameters of PPS.

Parameter	Value	Test Method	Parameter	Value	Test Method
Density/g·cm^−3^	1.50	ASTM D1505	Melting temperature/°C	280	ISO 75-2
Mold shrinkage/%	0.1–0.5	ASTM D1505	Tensile strength/MPa	160	ISO 527-2
Stretch film amount/GPa	11	ASTM D1505	Elongation/%	2.5	ISO 527

**Table 6:** Parameters of the injection molding machine.

Screw Diameter/mm	Metering Zone Length/mm	Injection Volume/g	Maximum Injection Pressure/MPa	Maximum Injection Speed/mm·s^−1^	Maximum Mold Thickness/mm	Maximum Clamping Force/t	Nozzle Hole Diameter/mm
28	140	110	290	300	500	180	2.5

**Table 7:** Injection process parameters.

Process Parameters	Parameter Value	Process Parameters	Parameter Value
Melting temperature/°C	305	Packing pressure/MPa	5
Mold temperature/°C	120	Packing time/s	5
Injection speed/mm·s^−1^	125	Injection delay/s	10
Back pressure/MPa	1	Cooling time/s	20

## Data Availability

This study did not report any data.
